# Race, Poverty, and Potential Exposure of Middle-School Students to Air Emissions from Confined Swine Feeding Operations

**DOI:** 10.1289/ehp.8586

**Published:** 2005-11-10

**Authors:** Maria C. Mirabelli, Steve Wing, Stephen W. Marshall, Timothy C. Wilcosky

**Affiliations:** 1 Department of Epidemiology, School of Public Health,; 2 University of North Carolina Injury Prevention Research Center and; 3 Department of Orthopedics, School of Medicine, University of North Carolina–Chapel Hill, Chapel Hill, North Carolina, USA; 4 Environmental Health and Epidemiology Program, RTI International, Research Triangle Park, North Carolina, USA

**Keywords:** adolescent health, children’s health, confined swine feeding, environmental epidemiology, environmental justice, industrial hog operations, school health

## Abstract

Previous studies suggest that airborne effluent from swine confined animal feeding operations (CAFOs) may affect the health and quality of life of adults and the prevalence of asthma symptoms among children. To investigate the extent to which public school students may be exposed to airborne effluent from swine CAFOs and to evaluate the association between schools’ demographic characteristics and swine CAFO exposures, we assessed the proximity of 226 schools to the nearest swine CAFO and conducted a survey of school employees to identify schools with noticeable livestock odor. We used publicly available information describing the enrollment of each school to assess the association between race and socioeconomic status (SES) and swine CAFO exposure. Odor from livestock was noticeable outside (*n* = 47, 21%) and inside (*n* = 19, 8%) school buildings. Schools with < 63% enrollment of white students and ≥47% of students receiving subsidized lunches at school were located closer to swine CAFOs (mean = 4.9 miles) than were the remaining schools (mean = 10.8 miles) and were more likely to be located within 3 miles of an operation than were schools with high-white/high-SES enrollment (prevalence ratio = 2.63; 95% confidence interval, 1.59–4.33). The prevalence of reported livestock odor varied with SES (low SES, 25%; high SES, 17%). These analyses indicate that the potential for in-school exposure to pollution arising from swine CAFOs in North Carolina and the environmental health risks associated with such exposures vary according to the racial and economic characteristics of enrolled students.

Confined animal feeding operations (CAFOs) house large numbers of animals, flush animal wastes into open-air waste pits, and apply partially decomposed wastes to land, releasing pollutants into soil, air, and water (National Research Council 2003). Odor and local air pollution—including ammonia (Reynolds et al. 1997; Subramanian et al. 1996; U.S. Environmental Protection Agency 2002), hydrogen sulfide (Reynolds et al. 1997), methane (Sharpe and Harper 1999), residues of veterinary antibiotics (Hamscher et al. 2003), total bacteria (Radon et al. 2001), fungi (Radon et al. 2001), and endotoxin (Reynolds et al. 1997)—arise from CAFO buildings and waste pits and are of particular concern to CAFO neighbors because of their documented impacts on the health and quality of life of livestock farm workers and neighbors (Cole et al. 2000; Merchant et al. 2005; Schiffman 1998; Schiffman et al. 1995; Thu et al. 1997; Vogelzang et al. 1999, 2000; Wing and Wolf 2000). A study of the mental and physical health of swine CAFO neighbors in Iowa found elevated rates of respiratory symptoms among CAFO neighbors compared with residents not living near livestock production (Thu et al. 1997). One study of swine CAFO neighbors in North Carolina reported negative impacts of odor on tension, depression, and anger among individuals living near operations (Schiffman et al. 1995), and another in North Carolina reported “increased occurrences of headaches, runny nose, sore throat, excessive coughing, diarrhea and burning eyes” and decreased quality of life among residents living near swine CAFOs (Wing and Wolf 2000). In an investigation of possible stress-mediated impacts on immune function, swine CAFO neighbors had lower average concentration and secretion of salivary immunoglobulin A during periods of moderate to high odor (Avery et al. 2004). Among children, increased prevalence of asthma symptoms has been associated with proximity to swine CAFOs (Chrischilles et al. 2004; Merchant et al. 2005).

In the United States, race and socioeconomic status (SES) are closely intertwined and have been widely associated with health, including chronic disease morbidity and mortality (Borrell et al. 2004; Roux et al. 2001; Winkleby et al. 1998), infectious diseases (Centers for Disease Control and Prevention 2005), immunization (Egede and Zheng 2003), health care services (Gaskin and Hoffman 2000; Monheit and Vistnes 2000; Weinick et al. 2000), and environmental exposures (Evans and Kantrowitz 2002; Guidry and Margolis 2005; Northridge et al. 2003). Swine CAFOs are disproportionately located in communities of color and regions of poverty (Edwards and Ladd 2000; Wilson et al. 2002; Wing et al. 1996, 2000) and are thus located among populations that may be more susceptible to the airborne exposures and more likely to experience detrimental health consequences of such exposures (Bradley and Corwyn 2002; Williams and Jackson 2005). The literature published to date about health impacts of CAFO-related exposures focuses on health impacts of exposures among adults; however, knowledge about the growth and development of the human respiratory tract suggests that the children in these exposed communities may be at increased risk of respiratory health effects because of their size, behavior, and developmental stage (Dietert et al. 2000; Kim et al. 2004; Peden 2000).

In light of recent research about health effects of CAFO-related exposures, children’s susceptibility to environmental pollutants, and concern about the conditions of school buildings, we sought to assess the extent to which adolescents attending public schools may be exposed to swine CAFO emissions. Based on a large sample of public schools in North Carolina, we estimated potential exposure using both record-based and survey-based exposure indices and examined racial and economic differences in potential exposure.

## Materials and Methods

During the 1999–2000 school year, seventh-and eighth-grade students from 499 public schools in North Carolina participated in a statewide school-based survey designed to assess the prevalence of asthma-related symptoms among adolescents (North Carolina Department of Health and Human Services 2001; Sotir et al. 2003). During the 2003–2004 school year, we conducted a follow-up survey of employees in the participating schools to collect information about environmental health conditions inside the schools and for an evaluation of the relationship between sources of environmental pollution located near schools and students’ self-reported respiratory health symptoms. From the 499 participating schools and based on the aims of our study, we excluded 160 schools from further data collection because of school locale and level of participation in the asthma survey. Specifically, we excluded schools located in counties with no swine CAFOs and none in neighboring counties (*n* = 45), schools with < 25 students surveyed (*n* = 34), schools located within 5 miles of a state border (*n* = 17), schools physically located within a city with population > 100,000 (*n* = 61), and schools that had closed or relocated to a new building since the 1999–2000 school year (*n* = 10). The remaining 339 schools composed our final target population of public schools.

We used publicly available records about the geographic positions of schools (North Carolina Center for Geographic Center and Analysis 2002) and swine CAFOs (Wing et al. 2000, 2002) to generate location-based estimates of in-school exposure for each school. We calculated distance to the nearest operation using the formula given by Goldberg et al. (1999) and categorized proximity as within or beyond 3 miles of the nearest operation. A 3-mile radius was selected as a suitable zone of potential exposure because elevated prevalence of asthma has been reported among children attending schools within 3 miles of a swine CAFO (Mirabelli et al., in press). Furthermore, although previous studies about the impacts of swine CAFOs on health and quality of life use a 2-mile radius (Thu et al. 1997; Wing and Wolf 2000), odors are sometimes reported at distances > 2 miles, and a radius of 3 miles yields a more balanced distribution of schools in our data. Swine CAFOs typically store animal waste in open waste pits, whereas other types of livestock operations in North Carolina tend not to employ such practices. Details about the locations of operations not using this liquid waste management system are not publicly available and could not be included in these analyses.

For a second metric of in-school exposure, we conducted a four-page, 21-item pencil-and-paper–style survey about environmental health conditions inside and surrounding the school buildings. In October 2003, we mailed the surveys to school principals and asked each to distribute four surveys to potential respondents in the following jobs: administrator, teacher, maintenance or custodial staff, and school nurse or health care provider. During a 9-month survey collection period, respondents from 267 (79%) of the surveyed schools returned 801 of the 1,632 surveys, whereas the remaining schools either actively (*n* = 1) or passively (*n* = 71) declined to participate. After receiving completed surveys, we excluded two additional schools based on updated information about the location of one school and because respondents from another school indicated that the school had closed and reopened in a new building since the 1999–2000 school year.

Respondents were asked whether odors from livestock farms were noticeable outside or inside the school buildings never, once per month or less, two to three times per month, about once per week, or more than once per week, and were asked to rate the odor, at its worst, on a five-point scale: 1, very faint; 2, faint; 3, moderate; 4, strong; 5, very strong. We assigned an odor rating of zero for respondents who indicated that they never noticed livestock farm odor at the school, and we created final school-level exposure indicator variables based on whether any survey respondent reported ever noticing livestock farm odor outside or inside the school building. For schools with livestock odors reported by any respondent, the odor ratings assigned to that school and used in the analyses are averages of the ratings provided by all survey respondents for the school. Because of publicity about the effects of industrialized swine production in North Carolina, we were concerned that survey questions specifically about swine CAFOs would cause response bias; therefore, respondents were asked about livestock odor in general. Public concern about odor from swine CAFOs has generated more reports to the health department than have other types of livestock operations in the state (Cline JS, personal communication). However, survey respondents did report odor from other livestock, primarily poultry; this was sometimes noted as a comment on the survey form. To avoid misclassifying these schools as being exposed to swine CAFO odor, we excluded from analysis 39 schools located > 5 miles from a swine CAFO for which respondents indicated the presence of livestock odor. Our final population for analysis was 226 public schools. Approximately 11% (145,704 of 1,315,363) of all students in North Carolina were enrolled in our population of schools during the 2003 school year.

To assess survey response within demographic and economic categories, we used data from the State of North Carolina (National Center for Education Statistics 2004; North Carolina Department of Public Instruction 2003) describing each school’s racial and ethnic composition and enrollment in the National School Lunch Program, used here as a proxy for SES. Students participating in the National School Lunch Program receive lunches for free or at reduced price, with the level of subsidy determined by the income of each child’s family. Children from families with incomes ≤130% or between 130 and 185% of the poverty level are eligible for fully or partially subsidized lunches, respectively (Child Nutrition and WIC Reauthorization Act 2004; U.S. Department of Agriculture 2004). We classified schools into race and SES categories using the median values of white enrollment (median, 63%) and subsidized lunch (median, 47%). The resulting matrix of race and economics was used to identify schools as high white/high SES (96 schools), low white/high SES (16 schools), high white/low SES (18 schools), and low white/low SES (96 schools).

We assessed the association between the race, economics, and both metrics of school-based swine CAFO exposure using binary regression in a log-linear model to estimate the prevalence of the exposures. Regression models were adjusted for rural school locale using data from the National Center for Education Statistics (2004), which uses information about proximity to metropolitan areas and population size and density to assign a locale code to each school. We categorized schools as rural if they were identified as “not within a consolidated metropolitan statistical area (CMSA) or metropolitan statistical area (MSA) and designated as rural” or “within a CMSA or MSA and designated as rural.” All remaining categories, including location within large or mid-size central cities, urban locations, or small towns with populations of at least 2,500, were categorized as nonrural. All independent variables in the models are school-level variables, and the resulting measures of association are prevalence ratios (PRs). We used SAS statistical software (version 8.2; SAS Institute Inc., Cary, NC) for all analyses.

## Results

**Figure 1:**
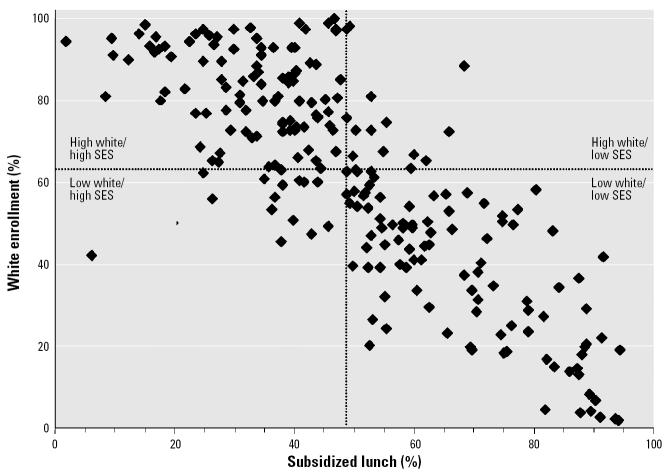
Distribution of white race and economic disadvantage in 226 public schools in North Carolina. Percentages are based on the population students enrolled during the 2003–2004 school year identified as white, non-Hispanic, and receiving subsidized lunches through the National School Lunch Program.

Across the 226 schools, mean enrollments of black and white students, respectively, were 26% and 63%. The mean enrollments of Asian students (< 1%), Hispanic students (3%), and Native-American students (< 1%) were low, and none of the schools had majority enrollment of Asian or Hispanic students. The percentage of enrolled students receiving fully or partially subsidized lunches was highly correlated with white, non-Hispanic enrollment (Figure 1).

**Figure 2:**
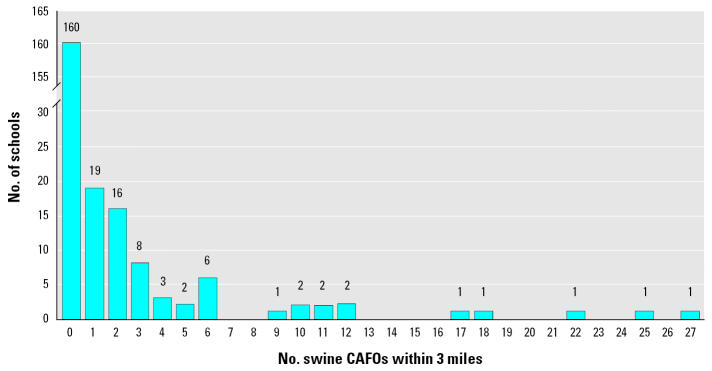
Number of schools by number of swine CAFOs within 3 miles.

**Figure 3:**
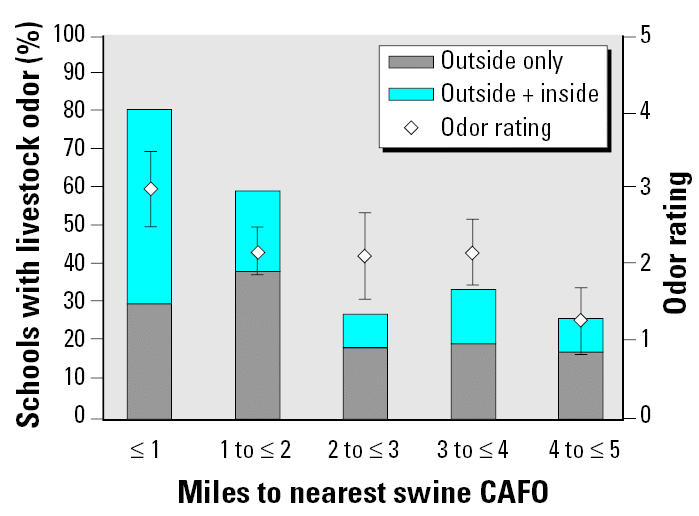
Percentage of schools with noticeable livestock odor and mean ± SE odor ratings for schools with reported odor, by distance between the school and the nearest swine CAFO.

For the 226 schools, distances between schools and the nearest swine CAFO ranged from 0.2 to 42 miles (mean ± SE, 8.3 ± 0.5), and mean distances increased across tertiles of white enrollment (low, 4.9; medium, 7.0; high, 12.7 miles) and SES (low, 4.6; medium, 8.4; high, 12.1 miles). Sixty-six (66) schools were located within 3 miles of one or more operations (Figure 2). Livestock odor was reported outdoors at 47 (21%) of the surveyed schools. In 19 schools (8%), the livestock odor was noticeable indoors, including in classrooms and hallways of the school buildings and in temporary, portable classroom buildings. Overall, the average livestock odor rating was 2.2 (SE = 0.2), which corresponds to an odor rating between “faint” and “moderate” on the scale used for the survey. The average rating of odor at schools with odor noticeable inside the school building was 2.8 (SE = 0.3). The percentage of schools reporting livestock odor and ratings of the strength of the odor each decreased with increasing distance to the nearest swine CAFO (Figure 3). The percentage of schools located within 3 miles of a swine CAFO was lowest (16%) in high-SES schools. A similar percentage (17%) was observed when exposure was considered using reported livestock odor.

Table 1 shows estimates of the relationship of race and SES with distance to the nearest swine CAFO. Having a swine CAFO within 3 miles was most prevalent in schools with low-white/low-SES enrollment [PR = 2.93; 95% confidence interval (CI), 1.79–4.80] compared with schools in the highest category of white enrollment and SES. Restricting the outcome to school location within 2 miles (*n* = 44) showed a similar trend of higher prevalence among low SES schools. A swine CAFO within 2 miles was more prevalent in schools with low-white/low-SES enrollment (*n* = 26; PR = 2.62; 95% CI, 1.38–4.97), high-white/low-SES enrollment (*n* = 5; PR = 2.43; 95% CI, 0.97–6.06), and low-white/high-SES enrollment (*n* = 2; PR = 1.39; 95% CI, 0.34–5.71) compared with schools with high-white/high-SES enrollment (*n* = 11). When exposure was considered using survey-based reports of livestock odor, the highest prevalences of noticeable odors outside or inside the school buildings were in schools with low SES enrollment (high white/low SES: *n* = 5, 28%; low white/low SES: *n* = 23, 24%), and the lowest prevalence of such odor was observed in schools with high-white/high-SES enrollment (*n* = 16, 17%) (Table 2). The mean (± SE) odor rating declined across tertiles of percent white, non-Hispanic enrollment (low, 2.1 ± 0.3; medium, 2.5 ± 0.4; high, 1.9 ± 0.4) and SES (low 2.4 ± 0.3; medium, 2.1 ± 0.3; high, 2.0 ± 0.5).

By excluding 39 schools for which survey respondents reported livestock odor, but located beyond 5 miles of a swine CAFO, we intended to reduce misclassification of schools located near nonswine CAFOs. Among the excluded schools, 33 had high enrollments of white students (high white/high SES, 25 schools; high white/low SES, 8 schools). Inclusion of these 39 schools approximately doubled the prevalence of reported odor (outside or inside, 34%; outside only, 18%; outside and inside, 19%) and resulted in marked attenuation of the effect of low white enrollment (outside or inside: PR = 0.89; 95% CI, 0.59–1.32; outside only: PR = 0.88; 95% CI, 0.48–1.61; outside and inside: PR = 0.86; 95% CI, 0.45–1.64).

## Discussion

In 2002, there were approximately 56,000 crop and livestock farms in North Carolina, and nearly 30% of the state’s land was used for agricultural production, including the cattle, hog, and poultry industries that significantly contribute to the state’s agricultural economy (North Carolina Department of Agriculture and Consumer Services 2003). Previous research about the presence of swine CAFOs shows a disproportionately high concentration of the industry in communities of color despite the declining number of black farmers in the southeastern United States (Wilson et al. 2002; Wing et al. 1996, 2000). In this study we examined the relationship of the racial and economic characteristics of students enrolled in public schools in North Carolina with estimated exposure to airborne effluent from nearby swine CAFOs and found that economic disadvantage was associated with proximity to the nearest swine CAFO and with strength of the odor. These findings suggest that swine CAFO emissions and any inhalable exposures, including odorant and nonodorant chemicals and respirable organic dusts, that correlate with odor disproportionately affect a population of children and adults who, regardless of their livestock-related exposures, may be predisposed to asthma-related health outcomes and other illnesses for reasons largely attributable to their economic disadvantage (Gee and Grimpayne-Sturgesalt 2004).

Odorous plumes arising from livestock farms contain a variety of gaseous and particulate elements, including inhalable dusts, bacteria, mold, hydrogen sulfide, ammonia, methane, pharmaceutical residues, and animal dander (Reynolds et al. 1997; Subramanian et al. 1996). In this study, the composition of the air present when livestock-related odors were reported and the specific agents responsible for the odor are both unknown. Without information about the extent to which odorous plumes from CAFOs contain respiratory irritants or odorants capable of inducing health effects (Shusterman 1992), we cannot draw conclusions about exposures relevant for respiratory health of the enrolled students, school employees, or neighbors. However, livestock-related odor at public school buildings indicates the presence of airborne livestock effluent beyond the agricultural land from which it arose and in the surrounding community. Reports of livestock odor outside and inside school buildings raise concern not only about health risks resulting from swine CAFO effluent but also about educational and behavioral consequences such as classroom disruptions that might occur when livestock odor reaches the classroom, anxiety associated with the students’ and staff members’ inability to avoid the odor or change their environments, and concerns or precautions for students who have a history of acute respiratory reactions. Our results clearly suggest that livestock odor is a more common problem for schools with lower SES enrollment. Livestock odors at public schools, particularly those in economically disadvantaged areas, may have broad implications for schools and communities if such schools are unappealing to new teachers and staff or if odors affect the retention of current employees, influence parent and volunteer involvement, or affect the use of school facilities for recreational and community purposes.

Because nonodorous pollutants arising from swine CAFOs may also be present in these communities, our analysis included distance as a measure of potential exposure to airborne swine CAFO effluent. Overall, we observed increased frequency of swine CAFOs near schools with above-median enrollment in the National School Lunch Program. Distances between schools and swine CAFOs were estimated using publicly available data about the locations of public school buildings and hog operations that raise more than 250 animals using a liquid waste management system. Smaller confinement-based operations, smaller “family farms,” and confined livestock operations that produce chickens, turkeys, or other animals are not included in our distance comparisons but may be included in reports of odor from livestock farms. We excluded 39 schools for which respondents reported livestock odor but that were located > 5 miles beyond a swine CAFO. Inclusion of the excluded schools approximately doubled the prevalence of reported odor, and the effect of low white/low SES, compared with high white/high SES, changed from elevated risk to reduced risk in each of the three livestock odor models, suggesting that schools with high white enrollment are disproportionately exposed to odors from other types of livestock operations. Among the excluded schools, 13% (*n* = 5) returned surveys with specific mention of livestock odor from poultry, compared with 3% (*n* = 7) of the schools included in our main analysis. In both populations of schools, reports of poultry odor were more common among schools with ≥63% enrollment of white students (excluded schools, 15%; schools in main analysis, 4%) than among schools with < 63% enrollment of white students (excluded schools, 0%; schools in main analysis, 2%); a less pronounced division was observed across categories of SES. Information about odor from poultry operations was not directly solicited on our surveys, so our supposition that poultry operations are located near schools with higher white enrollment, based on the demographics of schools with survey-reported poultry odor, is uncertain. We were unable to evaluate the proximity of schools to poultry operations because few poultry operations in North Carolina require government-issued liquid waste management permits from which location data can be abstracted. The State of North Carolina does not currently release information about the locations of poultry CAFOs because of state regulations about confidentiality of agricultural data (State of North Carolina 2002).

### Study limitations.

Our survey-based reports of livestock odor are vulnerable to several sources of potential bias. If respondents at schools with higher enrollment of white students are more likely to report livestock odor on our survey than are respondents at schools with higher nonwhite enrollment, then this finding may be the result of biased survey response. If ventilation or window use correlate with enrollment, then differences in the odor reports may be due to differences in indoor odor levels. School SES may be correlated with the size, age, technology, or other features of livestock operations that affect odor. And, although in the distance-based analyses we categorized proximity to a swine CAFO as within 3 miles of at least one swine CAFO, the number of swine CAFOs located near a school and the distances and geographic directions between the school and each of the nearby swine CAFOs are each reflected in the survey-based estimates of swine CAFO exposure. The surveys provided an estimate of total exposure, whereas the analysis based solely on distance may have underestimated the burden of exposure on schools located near more than one swine CAFO. These components of exposure would be important to consider in an assessment of health impacts of swine CAFO–related exposures.

Our sample size was determined largely by whether employees in each of the surveyed schools participated in our environmental health survey. If the presence of livestock odor at the school or the presence of the livestock industry in the community systematically influenced employees’ decisions to complete and return surveys, then our sample of schools may not be representative of the surveyed population. If embarrassment, denial, or exaggeration of the odor problem affected respondents’ odor ratings, or if respondents’ adaptation to the odor affected their ratings, then the distribution of odor reported on our surveys may not reflect the presence of odorant chemicals in this population of schools. For example, if respondents in farming communities and who routinely smell livestock odor rate the odor as less severe than do survey respondents who are not routinely exposed outside of the school, then the exposures of more exposed schools may be underestimated in these data.

To assess potential bias in survey response, we evaluated school-level survey participation and found that response rates increased across tertiles of increasing percent enrollment of white, non-Hispanic students (< 51% white, 75% participation; 51% to < 78%, 77%; ≥78%, 85%). Lower participation among schools with larger nonwhite populations may reflect a broad pattern of nonparticipation in research activities initiated by predominantly white institutions (Corbie-Smith et al. 1999, 2004; Gamble 1993; Shavers-Hornaday et al. 1997). Among participating schools, our classification of the presence of livestock odor based on employees’ responses to the survey question may have introduced additional bias in our results. We mailed more than one survey to each school and received up to seven completed surveys per school; for each survey question, we assigned the exposure to a school if any respondent indicated the presence of livestock odor at the school. Consequently, our exposure assignments were sensitive to the number of surveys completed and returned from each school. With each additional survey returned from a single school, and with each additional respondent providing a new opportunity for the school to be classified as exposed, the likelihood of a school’s classification as having noticeable livestock odor increased. To assess the impacts of our use of all survey responses and our method of classifying exposure, we estimated the effect of race and economic characteristics on livestock odor using data from one randomly selected survey from each of the participating schools. We repeated this sampling 50 times to generate a range of estimates. On repeated sampling and estimation of the effect of race and economics on any noticeable livestock odor, variation in PRs was low, with 76% (38 of 41) of low-white/high-SES estimates, 80% (40 of 50) high-white/low-SES estimates, and 42% (21 of 50) of low-white/low-SES estimates being closer to the null than the results we report.

## Conclusions

Our results provide evidence that North Carolina’s swine CAFOs are located closer to schools enrolling higher percentages of non-white and economically disadvantaged students and that livestock odor is a more common problem for schools with lower SES enrollment. By considering the environmental exposures that adolescents attending school near the facilities may experience, our findings support and extend previous research about the density of swine CAFOs in nonwhite and poor communities (Wilson et al. 2002; Wing et al. 2000), and the association between environmental exposure and race and poverty in communities located near industrial sources of air pollution (Perlin et al. 1999). Understanding the vulnerability of populations bearing the burden of swine CAFO exposures is of public health importance because of the health risks associated with swine CAFOs and swine odor in other studies (Avery et al. 2004; Merchant et al. 2005; Thu et al. 1997; Wing and Wolf 2000) and the likelihood that hazardous air pollutants arising from swine CAFOs affect the health of children in similar ways. Our findings may have implications for school personnel, particularly those in economically disadvantaged communities, who are concerned about this common exposure and its potential impact on adolescents’ respiratory health and should be used to address existing racial and economic disparities in exposure to environmental health hazards.

## Figures and Tables

**Table 1:** Associations between distance to the nearest swine CAFO and public school enrollment in North Carolina.

		Distance to nearest swine CAFO		
			≤3 miles	
Enrollment^a^	All schools (no.)	> 3 miles No. (%)	No. (%)	PR (95% CI)^b^
All schools	226	160 (70.8)	66 (29.2)	
High white/high SES	96	80 (83.3)	16 (16.7)	1.00
High white/low SES	18	12 (66.7)	6 (33.3)	1.95 (0.90–4.25)
Low white/high SES	16	14 (87.5)	2 (12.5)	0.95 (0.24–3.72)
Low white/low SES	96	54 (56.3)	42 (43.8)	2.93 (1.79–4.80)

a Enrollment categories: high white, ≥63% enrollment of white, non-Hispanic students; low white, < 63% enrollment of white, non-Hispanic students; high SES, < 47% of students receiving free or reduced price lunch at school; low SES, ≥47% of students receiving free or reduced price lunch at school.

b Adjusted for rural school locale.

**Table 2:** Associations between noticeable livestock odor and public school enrollment in North Carolina.

			Outside or inside		Outside only		Outside + inside	
Enrollment^a^	All schools (no.)	No odor No. (%)	No. (%)	PR (95% CI)^b^	No. (%)	PR (95% CI)^b^	No. (%)	PR (95% CI)^b^
All schools	226	179 (79.2)	47 (20.8)		28 (12.4)		19 (8.4)	
High white/high SES	96	80 (83.3)	16 (16.7)	1.00	9 (9.4)	1.00	7 (7.3)	1.00
High white/low SES	18	13 (72.2)	5 (27.8)	1.63 (0.70–3.80)	4 (22.2)	2.42 (0.86–6.84)	1 (5.6)	0.89 (0.12–6.62)
Low white/high SES	16	13 (81.3)	3 (18.8)	1.44 (0.48–4.30)	2 (12.5)	1.87 (0.45–7.79)	1 (6.3)	1.12 (0.15–8.49)
Low white/low SES	96	73 (76.0)	23 (24.0)	1.58 (0.90–2.78)	13 (13.5)	1.63 (0.74–3.61)	10 (10.4)	1.66 (0.66–4.15)

a Enrollment categories: high white, ≥63% enrollment of white, non-Hispanic students; low white, < 63% enrollment of white, non-Hispanic students; high SES, < 47% of students receiving free or reduced price lunch at school; low SES, ≥47% of students receiving free or reduced price lunch at school.

b Adjusted for rural school locale.
